# The King's Hospital Fund for London and St. George's Hospital

**Published:** 1911-12-23

**Authors:** 


### THE KING'S HOSPITAL FUND FOR LONDON AND ST. GEORGE'S HOSPITAL.

### The Position Made Clear.

No doubt our readers will have noticed that in paragraph 2 of the report of the Distribution Committee of the King's Fund, which we published on page 289 of The Hospital for December 16, reference is made to the omission of the name of St. George's Hospital from the list of awards for 1911. We have received the following letter from Mr. A. William West, Chairman and Treasurer of St. George's Hospital, on this subject.

### The View of St. George's Hospital.

Dear Sir,—In the report of the meeting of the King Edward's Hospital Fund for London, the following paragraph appeared :—

"Among the institutions which do not appear in this year's list is St. George's Hospital, the Governors having withdrawn their application as the result of the decision of the Fund on the question of the amounts which could properly be paid to the Medical School in respect of the work done for the hospital. The grant of £2,000 which was reserved last year lapses, the conditions attached to it by the Council of the Fund not having been fulfilled."

The Governors of St. George's Hospital have always endeavoured to work in harmony with the King's Fund, and much regret the difference of opinion upon a matter of principle, which they consider to be of such importance as to justify their refusal to comply with the demands of the Fund. The Governors maintain that no money whatever is paid by the hospital to the Medical School for the purposes of education.

The sum referred to has been expended upon pathological and bacteriological work done entirely for the patients of the hospital; in other words, the money is spent in giving the patients of St. George's Hospital the benefit of the latest scientific improvements in diagnosis and treatment.

In previous years, when the same money was paid out of a discretionary fund, no objection was made by the King's Fund, but in such a case the accounts of the Hospital are fallacious to the extent that the sum paid out of a discretionary fund cannot appear in the final calculation of the cost per bed.

The Governors of St. George's Hospital believe that all moneys paid for the benefit of patients should be paid out of the general funds and published in the accounts.

Feeling sure that no good can result either to the King's Fund or St. George's Hospital by the public discussion of the questions involved, the whole circumstances of the case are not now entered into, but a statement can be obtained by those who are interested upon application to the Secretary.—I am, yours faithfully,

A. William West,
Chairman and Treasurer.

St. George's Hospital,

S.W., December 13.

### The Decision of the King's Hospital Fund.

To enable our readers to understand the position exactly we take the following paragraph from the report of the Distribution Committee for the year 1910, which was adopted at the meeting of the Council of the King's Fund on December 14, 1910:—

Hospitals and Medical Schools.—The Case of St. George's.

In the case of St. George's Hospital, the Committee have had under their special consideration the appearance, in the general accounts of the hospital for 1909, of certain payments to the school which were stated to be for work done for the hospital, and which were formerly paid out of a discretionary fund. The inquiries made by the committee as to the legitimacy of this change confirmed the statement in the annual report of the hospital that the payments in question were made on account of definite work done, chiefly pathological and bacteriological examinations, in connection with the actual treatment of patients. That is, the payments were for the kind of work which is described in Appendix III. of the report of Sir Edward Fry's committee as work which would have to be paid for in any case, and which would have to be taken into account in assessing the payments and receipts on either side. Such work, moreover, differs entirely from the general benefits, described in paragraphs 13 to 22 of the report as being conferred by medical schools on hospitals, and as being fairly set off against the benefits conferred by the hospitals on the schools. The opinion of the Fund, that, on the principles laid down by Sir Edward Fry's committee, specific payments for such definite work done in respect of the treatment of patients, whether made to the school or to any other agency, could be charged to the general funds of the hospital, while no other payments to the school could be so charged, was notified to St. George's, in reply to a question from the hospital, by the executive committee in March 1909. But on looking into the method by which the amount fairly chargeable to the hospital on account of this work had been arrived at, the Distribution Committee came to the conclusion that the apportionment of the expenses of the laboratories as between the treatment of patients and medical education required investigation, and they have invited the hospital to submit further details in order that the committee may satisfy themselves that the payment made to the school bears a proper proportion to such definite work done for the hospital.

In the meantime they recommend that the grant to the hospital this year should be retained until the Fund is satisfied that the financial relations between hospital and school are in accordance with the principles intimated by the Fund in March 1909, and that the general account of the hospital in respect of 1909, as well as 1910, is clear from any payment on account of medical education.

It will be noticed in the paragraph Mr. West quotes from the report of the Distribution Committee for 1911 "that the grant of £2,000, which was reserved last year, lapses, the conditions attached to it by the Council not having been fulfilled." From this it would appear that the King's Fund is not satisfied that at St. George's Hospital, "the financial relations between hospital and medical school are in accordance with the principles intimated by the Fund in March 1909, and that the general accounts of the hospital are clear from any payment on account of medical education."

